# Supplementary material for: The Unified Phenotype Ontology : a framework for cross-species integrative phenomics
Source: Genetics. 2025 Mar 6;229(3):iyaf027. doi: 10.1093/genetics/iyaf027 (PMC11912833; doi:10.1093/genetics/iyaf027)
Supplement: iyaf027_Supplementary_Data [file iyaf027_supplementary_data.docx]

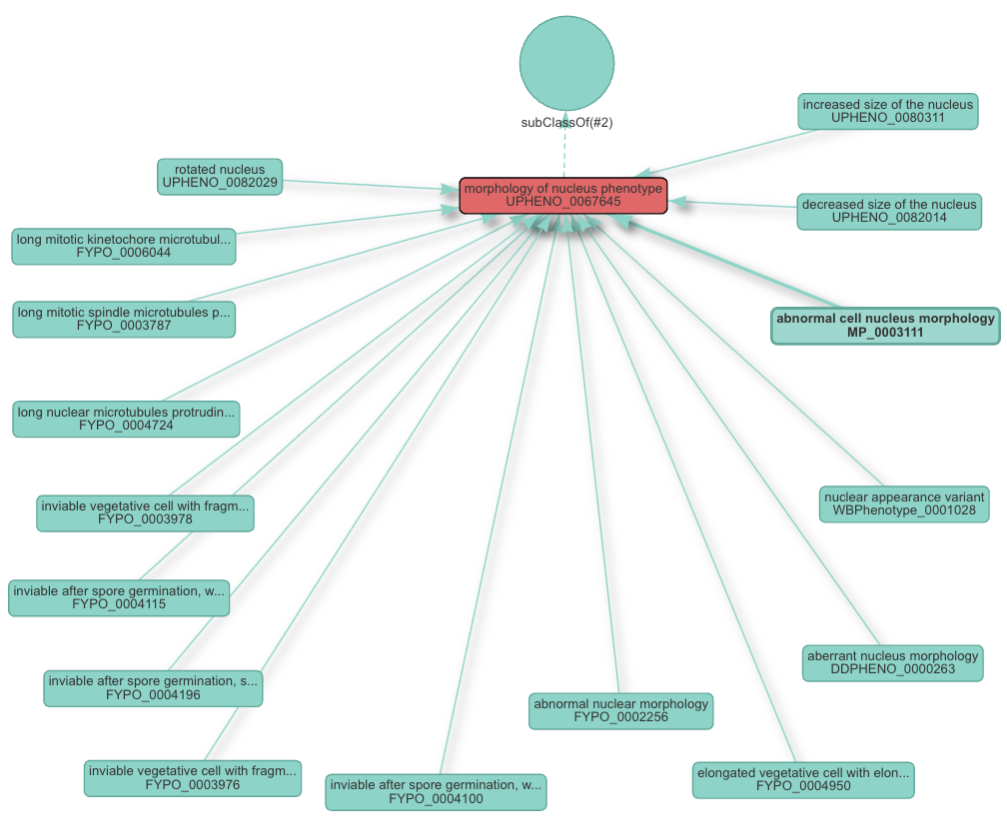


**Figure S1.** The uPheno term "UPHENO:0067645 morphology of nucleus phenotype" can group together analogous phenotypes from distantly related taxa, such as fission yeast (including "FYPO:0002256 abnormal nuclear morphology"), Dictyostelium discoideum (e.g. "DDPHENO:0000263 aberrant nucleus morphology"), nematodes (e.g. "WBPhenotype:0001028 nuclear appearance variant"),  and mammals (e.g. "MP:0003111 abnormal cell nucleus morphology"). Grouping together these phenotypes allows linking useful knowledge to help phenotype interpretation and prioritization.
